# Monitoring of Schmallenberg virus, bluetongue virus and epizootic haemorrhagic disease virus in biting midges in Germany 2019–2023

**DOI:** 10.1186/s13071-025-06913-w

**Published:** 2025-07-05

**Authors:** Sophie Zeiske, Helge Kampen, Franziska Sick, Oliver Dähn, Anja Voigt, Elisa Heuser, Martin Beer, Doreen Werner, Kerstin Wernike

**Affiliations:** 1 Friedrich-Loeffler-Institut, Federal Research Institute for Animal Health, Greifswald - Insel Riems, Germany; 2 Leibniz-Centre for Agricultural Landscape Research, Muencheberg, Germany

**Keywords:** Bunyavirus, Orbivirus, Schmallenberg virus, Bluetongue virus, Epizootic haemorrhagic disease virus, Insect vector, Prevalence, Biting midges, *Culicoides*, Monitoring

## Abstract

**Background:**

Schmallenberg virus (SBV) was first detected in Germany in 2011 and today has an enzootic status in Central Europe. It is transmitted by biting midges of the genus *Culicoides*, which have a high abundance in livestock farms. In addition to SBV, *Culicoides* are considered vectors of other viruses relevant to livestock such as bluetongue virus (BTV) and epizootic haemorrhagic disease virus (EHDV). Monitoring of midges and transmitted viruses is of veterinary importance because the resulting diseases may cause animal suffering and entail economic losses due to control measures such as vaccination or trade restrictions.

**Methods:**

To gain an overview of the prevalence of viruses in *Culicoides* vectors in Germany, a monitoring programme was established in 2018. From 2019 to 2023, biting midges were caught at 79 sites throughout the country, of which 511,788 were morphologically differentiated according to *Culicoides* species or subgenus and pooled accordingly. The nucleic acids extracted from 19,521 midge pools of up to 50 individuals were tested in real-time reverse transcription polymerase chain reactions (RT-PCRs) for the genomes of SBV, EHDV and BTV. The species in virus-positive pools were analysed with molecular biological methods to identify potential vector species.

**Results:**

Whereas no EHDV and BTV were detected, SBV was found in every year of the five monitored years. The minimum infection rate (MIR) of SBV in the tested pools ranged from 3.75 in 2022 to 135.47 in 2023. Most SBV RNA-positive pools were represented by the subgenus *Avaritia* (*C. obsoletus*, *C. scoticus*, *C. dewulfi* and *C. chiopterus*). To a lesser extent, SBV RNA was detected in pools of the subgenus *Culicoides* (*C. punctatus*, *C. pulicaris*, *C. lupicaris* and *C. selandicus*). Only one pool of another subgenus, namely *C. griseidorsum*, was found positive for SBV genome.

**Conclusions:**

The results from the monitoring programme confirm an enzootic circulation of SBV in the German *Culicoides* population during summer and autumn with varying infection rates between the years. The lack of detection of BTV in the midges may suggest a circulation of BTV at a low level. The absence of EHDV genome in biting midges is in line with the epidemiological situation in ruminants in Germany.

**Graphical Abstract:**

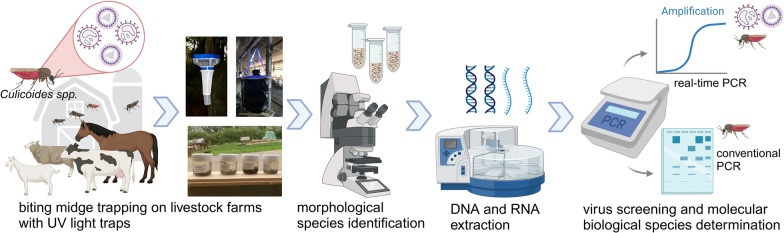

## Background

Schmallenberg virus (SBV) is an arbovirus of the genus *Orthobunyavirus*, which infects ruminants and is transmitted by biting midge species of the genus *Culicoides* (Diptera: Ceratopogonidae) [1]. Since its first emergence in 2011 in the west of Germany, SBV rapidly spread across the European continent [1, 2]. Recently, SBV has reached an enzootic status in Central Europe [3, 4]. Since 2011, SBV cases have been occurring every year in domestic ruminants in Germany with varying extent of virus detection between the years (source: German animal disease reporting system, *Tierseuchen-Nachrichtensystem* (TSN)). Occasionally, extensive virus circulation could be observed, resulting in a seroprevalence of up to 70%, and partly to 100%, in the affected livestock populations [5]. SBV causes mild or subclinical infections in ruminants such as cattle, sheep and goats [4, 6, 7]. The more severe problem is the infection of naïve pregnant dams, especially in early pregnancy [8]. When the virus is transmitted vertically, it can cause premature birth, stillbirth, abortions or malformations of the calves, lambs and goat kids [8, 9].

Further important viruses that are transmitted by *Culicoides* midges and affect domestic ruminants are the orbiviruses bluetongue virus (BTV) and epizootic hemorrhagic disease virus (EHDV) [10]. BTV occurs worldwide and can be differentiated into 24 classical and a number of “atypical” serotypes [11–13]. Several BTV serotypes and strains have been registered in Central Europe since its first emergence in 2006 [12, 14, 15]. The most recent introduction of a new serotype took place in 2023, when BTV serotype 3 (BTV-3) emerged in the Netherlands and subsequently spread to further Central European countries including Germany [16, 17]. Most domestic and wild ruminants are susceptible for BTV, but the severity of bluetongue disease depends on the affected ruminant species and the virus strain [13]. The BTV serotypes that emerged in Europe generally caused more severe disease in sheep than in cattle or goats [12]. Bluetongue disease is characterised by haemorrhagic fever with ulcerations, hyperaemia and oedemas especially of the facial mucosa, but it can also affect internal organs, causing breathing difficulties and death [18–21]. Furthermore, BTV may cause transplacental infections in pregnant cattle, goats and sheep leading to abortions or the birth of malformed fetuses [22–24].

Another representative of the orbiviruses that infects ruminants and is transmitted between its mammalian hosts by *Culicoides* biting midges is EHDV [25]. Similar to BTV, it affects the vascular system and causes haemorrhages and ulcerations. EHDV was first detected in North American white-tailed deer, which show severe clinical signs and high morbidities of up to 90% [10, 26, 27]. A wide range of other wild and domestic ruminants are susceptible to EHDV; however, the course of the disease can be quite variable, depending on the virus strain and the ruminant species [10]. It was assumed that most serotypes cause asymptomatic infections in cattle and sheep [28]. In 2022, EHDV serotype 8 was detected for the first time on the European continent, where it was found in Italy and Spain in cattle and sheep that showed BTV-like symptoms [29, 30].

Infections of domestic animals with SBV, BTV and EHDV cause massive economic damages to the livestock owners and the agricultural industry. The resulting diseases go along with direct costs for production loss, animal loss and veterinary treatment [31, 32]. Further, outbreaks of the diseases cause indirect costs for vaccination, if vaccines are available, trade restriction, diagnostics and surveillance [31, 32].

The genus *Culicoides* consists of around 1,350 species which have a body size of 1–3 mm and belong to the smallest haematophagous dipterans in the world [33–35]. They can be found in a wide range of habitats worldwide, except for the Arctic and Antarctic [35]. Specimens of the genus *Culicoides* can be identified by their wing patterns, morphological characters of the antenna, palpae and genital structures as depicted by Delécolle and Mathieu et al. for the European species [36, 37]. In the Palaearctic region, most *Culicoides* species belong the subgenus *Avaritia* Fox, followed by the subgenus *Culicoides* Latreille, and only a minor part belongs to other subgenera [38–41]. Due to the morphological uniformity of the females, biting midges of the subgenera *Avaritia* and *Culicoides* often cannot be identified to species level by morphological methods and, for practical reasons, are only assigned to subgenus, species group or species complex [42–44]. Species of the subgenus *Avaritia* frequently observed in Germany are *C. obsoletus*, *C. scoticus*, *C. dewulfi* and *C. chiopterus* [42, 45–49]. Based on the highly similar female morphology, *C. obsoletus* and *C. scoticus* are placed in the Obsoletus complex [50]. Widely distributed species of the subgenus *Culicoides* in Germany are *C.* *punctatus*, *C. impunctatus*, *C. deltus*/*lupicaris*, *C. newsteadi*, *C. grisescens* and *C. pulicaris* [42, 45–49]. Both in the subgenus *Avaritia* and in the subgenus *Culicoides*, haplotypes and cryptic species have been described, and there is increasing evidence that these genetic variants represent independent species [51–53].

The composition of the *Culicoides* fauna encountered in Germany can vary depending on the trapping location, environment and the time of the year, since the species differ in their distribution, host preference and behaviour [46, 54]. Since differences in vector competence occur, it is desirable to identify the *Culicoides* to species level. As reviewed by Purse et al. [55] vector competence was compared for several midge-borne viruses and different midge species. Several of the *Culicoides* species within one subgenus are demonstrated vectors while others are not [55]. Vector competence is defined as the ability of a biting midge to replicate and transmit a virus during a blood meal [56]. The premise for the vector competence is the full dissemination of the virus in the body of the biting midge and the replication in the salivary glands [57]. The appropriate approach of demonstrating vector competence and infectivity of *Culicoides* is the separate examination of the body and the head [56]. Since biting midges do not possess salivary gland barriers, the detection of virus in the head of a midge is deemed sufficient evidence for full dissemination and infectivity [57]. Vector competence for SBV was only shown for the species of the Obsoletus complex, *C. imicola* (subgenus *Avaritia*) and, with some restrictions, for *C. chiopterus* and *C. dewulfi* [38, 58, 59]. By contrast, it could still not be shown yet whether species of the subgenus *Culicoides* can replicate and transmit SBV or are just carriers of the virus; therefore, they keep being regarded as putative vectors [56]. In Europe, confirmed vectors of BTV are *C. imicola*, *C.* *scoticus*, *C. obsoletus*, *C. nubeculosus*, *C. impunctatus* and *C. pulicaris* [55, 60–64], although *C. nubeculosus* and *C. impunctatus* seem to play a minor role in the field [35, 55]. Additionally, *C. chiopterus*, *C. dewulfi* and *C. punctatus* are considered vector-competent for BTV [55, 65, 66]. Competent European vectors of EHDV are *C. imicola*, *C. obsoletus*, *C. punctatus* and *C. scoticus* [67–69].

In Germany, species of *Culicoides* can occur as a nuisance to humans and animals with high population densities inside and outside of livestock farms. In agricultural settings, there is a ready supply of moist environments and organic materials that serve as breeding substrates for female midges. Additionally, the female midges have access to blood meals from farm animals, which is usually essential for their egg production [70, 71]. The life span of adult midges is usually between 10 and 20 days [35]. In their short life, they stay mostly in a close distance of a few hundred meters to the breeding sites [35]. However, in several cases, biting midges can be spread over much greater distances, assisted by prevailing winds [72]. In temperate zones, the adult activity of biting midges is particularly high from late spring to early autumn [56], but a low number of adults is also active during the winter months and can be trapped inside and outside livestock buildings [42, 46].

The means by which SBV and BTV overwinter remain unclear [73, 74]; however, transovarial transmission was demonstrated recently for EHDV in an experimental study with *C. sonorensis* [75]. Evidence from infection studies is missing for SBV; however, detection of SBV in field-collected male or nulliparous female midges may indicate overwintering of the virus in immature stages of *Culicoides* [41, 76]*.*

Since 2011, cases of SBV in domestic ruminants have been reported annually all over Germany, though with variations in the number of reports between the years (source: TSN). In addition, the newly introduced BTV-3 has been spreading within the ruminant population since October 2023 (source: TSN). To gain an overview of the prevalence of the viruses in the *Culicoides* spp. in Germany, a monitoring programme was established. Since 2019, biting midges have been caught inside and outside livestock farm buildings at numerous locations all over Germany. They are classified according to *Culicoides* subgenus or species, using morphology and genetics. The *Culicoides* females are also investigated for the presence of SBV, BTV and EHDV genome.

## Methods

### *Culicoides* trapping

**Fig. 1:**
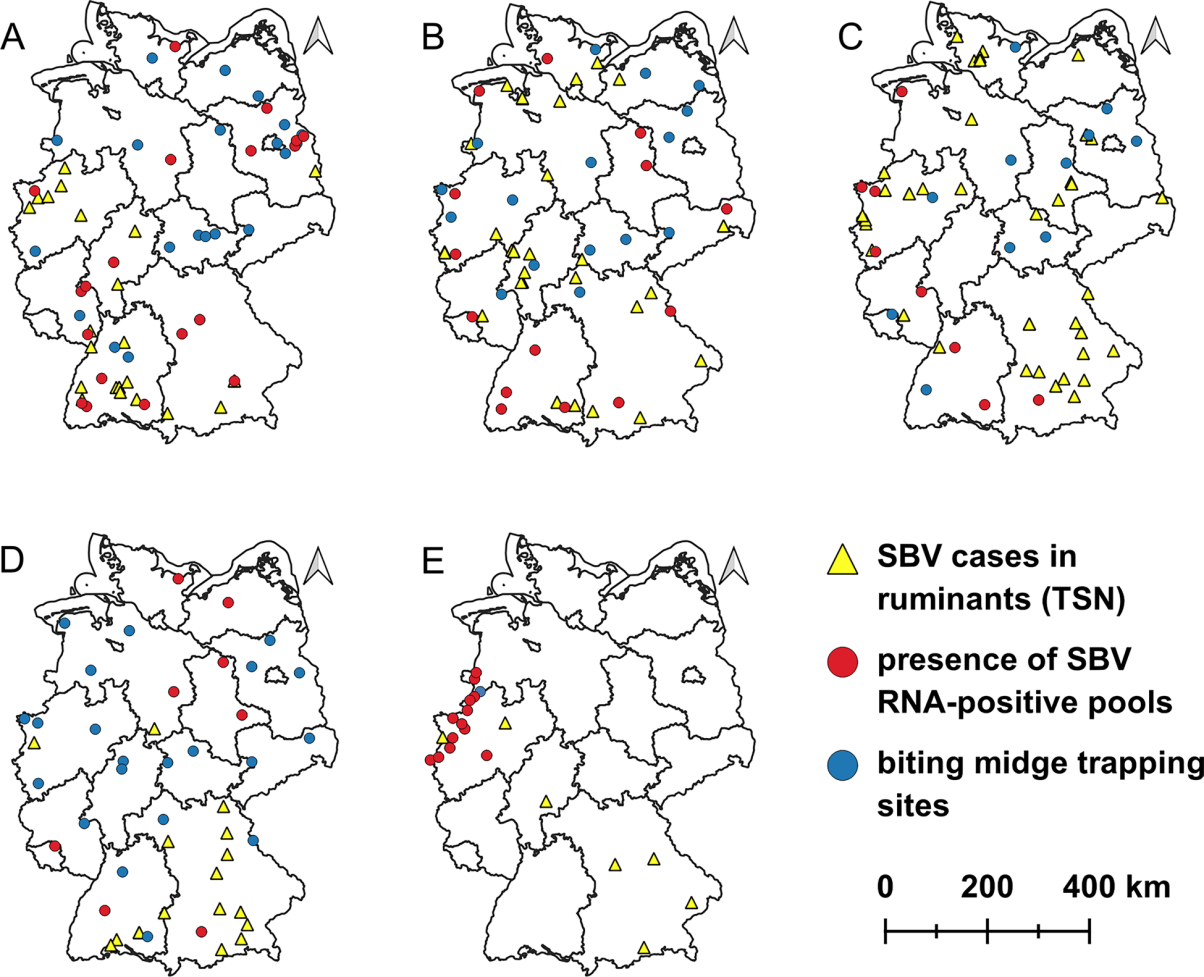
*Culicoides* trapping sites in Germany. Biting midges were caught from January 2019 to March 2020 (**A**), from April 2020 to March 2021 (**B**), from April 2021 to March 2022 (**C**), from April to October 2022 (**D**) and from September to November 2023 (**E**). Blue dots depict trapping sites with no SBV RNA-detection in the pools, red dots depict trapping sites with SBV RNA-positive pools and yellow triangles mark SBV cases in ruminants in each monitored year (German animal disease reporting system, TSN). Map of Germany was retrieved from Federal Agency for Cartography and Geodesy, data set Verwaltungsgebiete 1:2 500 000, Stand 31.12. (VG2500 31.12.) (https://gdz.bkg.bund.de/index.php/default/open-data/verwaltungsgebiete-1-2-500-000-stand-31-12-vg2500-12-31.html)**,** data license Germany– attribution–Version 2.0 (https://www.govdata.de/dl-de/by-2-0)

From 2019 to 2022, biting midges were collected all over Germany on farms keeping cattle, sheep, goats and/or horses by BG-Sentinal traps equipped with 12 V light-emitting diode (LED) ultraviolet (UV) light (Biogents AG, Regensburg, Germany). In 2019, *Culicoides* were caught at 37 sites, in 2020 at 30 sites, in 2021 at 19 sites and in 2022 at 29 sites (Fig. 1). The traps were placed both inside and outside of livestock housing structures, whenever possible within barns. If indoor placement was not feasible due to local conditions, the trap was positioned outdoors in sheltered locations (e.g. under roof overhangs or animal shelters on pasture), always as close as possible to the animals. ‘Inside’ thus refers to trap placement within a conventional barn building, while ‘outdoors’ refers to sheltered positions in close proximity to livestock but not enclosed. The traps were mounted at a height of 1.5–2.0 m. A polyester mesh fabric with a mesh size of 4 mm was used to prevent the capture of larger insects. Traps were operated once a week for 24 h, although the trapping day was selected independently at each site. Outdoor sampling was not carried out during adverse weather conditions such as rain or strong winds; however, no overall synchronization across sites took place. In all monitored years, the trapping was conducted from April to October. In addition, several farms participated in a winter monitoring during the months of November to March: 11 farms from January to March 2019, 16 farms in both 2019/2020 and 2020/2021 and 12 farms in 2021/2022.

**Fig. 2:**
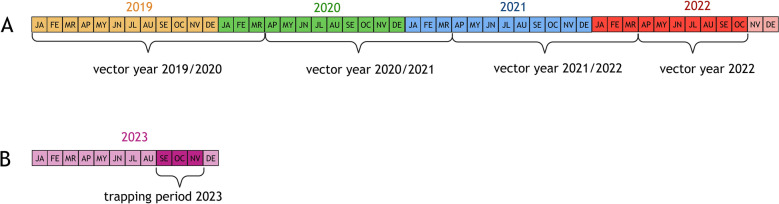
Definition of the trapping periods. **A** Definition of the vector years 2019/2020, 2020/2021, 2021/2022 and 2022. **B** Additional trapping period 2023. The coloured squares depict the months of the years (2019 yellow, 2020 green, 2021 blue, 2022 red, 2023 magenta). Months in which midges were trapped are coloured in a darker colour shade, and months in which no trapping was conducted are coloured in a lighter shade. *JA* January, *FE* February, *MR* March, *AP* April, *MY* May, *JN* June, *JL* July, *AU* August, *SE* September, *OC* October, *NV* November, *DE* December

In 2023, additional UV-light traps were placed on 13 livestock farms close to the Dutch border in the German federal states of North Rhine-Westphalia and Lower Saxony. From September 24 until November 12, the midges were collected daily at these places (Fig. 2B).

All insects were trapped directly into 80% ethanol, as provided in a beaker in the trapping net. Upon collection, samples were kept in the dark at room temperature. Biting midges remained in ethanol until nucleic acid extraction, i.e. from capture through storage and sorting.

### *Culicoides* identification

The insects were examined under a stereomicroscope (Leica model M205 C; Leica Microsystems GmbH, Wetzlar, Germany) to sort out by-catch and non-haematophagous midges. Only *Culicoides* biting midges were included in subsequent analyses, with female and male specimens being processed separately. The morphological differentiation of the female biting midges was done prior to pooling using the identification keys of Mathieu et al. [36] and Delécolle [37]. Because of morphological similarities, not all midges could be differentiated on species level and were therefore assigned to the subgenera *Avaritia* or *Culicoides*. Species belonging to other subgenera were provisionally assigned to their specific subgenera before genetic identification. Females were additionally categorised on the basis of visible blood-feeding status (blood-fed/non-blood-fed). Other parity-related classifications, such as nulliparous/parous or gravid, were not assessed. Blood-fed females were processed using the same virus screening protocol as non-blood-fed specimens.

For virus screening, midges were pooled with up to 50 midges according to the date and location of trapping and the determined subgenus or species. If more than 50 midges of a subgenus or species were caught on 1 day on a trapping location, they were pooled in several pools containing up to 50 midges. *Culicoides* specimens morphologically identified to species level were tested individually. Not all pools of female midges were tested for virus RNA, but several were stored as retention samples. Biting midge pools which tested positive for SBV, BTV or EHDV were subsequently analysed by polymerase chain reactions (PCR) for species included in the pool [51, 52].

### Nucleic acid extraction and virus screening by real-time RT-PCR

To prepare the biting midge pools for nucleic acid extraction, the ethanol was drawn off and the midges were dried overnight at room temperature. Thereafter, the pools were homogenised in 200 µl Lysis Buffer from the NucleoMag Vet Kit using the TissueLyser (Qiagen, Hilden, Germany) with a 5-mm steel bead at a frequency of 30 Hz for 3 min. Nucleic acid was extracted with the King Fisher 96 Flex purification system and the NucleoMag Vet Kit (Machery Nagel, Düren) according to the manufacturer’s instructions. All extracts were tested by previously described real-time reverse transcription polymerase chain reactions (RT-PCRs) for the presence of SBV [77] and BTV and EHDV RNA [78].

### Molecular identification of other *Culicoides* species

Barcoding of the cytochrome c oxidase subunit I (COI) gene was used to identify individual *Culicoides* that did not belong to the subgenera *Avaritia* or *Culicoides*. PCR amplification was performed with the forward primers PanCuli-COX1-025F [51] or PanCuli-COX1-211F, paired with the reverse primer PanCuli-COX1-727R, using an adapted protocol (54 °C annealing temperature) from Lehmann et al. [79]. PCR products were mixed with 2.5 µL of 6× DNA loading dye (Thermo Fisher Scientific, Dreieich, Germany) and analysed on 1.5% agarose gels containing 5 mg/mL ethidium bromide. Electrophoresis was conducted at 100 V for 50 min, and PCR bands were visualised using a ChemiDoc MP Imaging System (Bio-Rad, Feldkirchen, Germany). Amplicons of the expected size were excised and purified with the QIAquick Gel Extraction Kit (Qiagen).

DNA fragments were cycle-sequenced in one or both directions using the PCR primers and the BigDye Terminator v1.1 Cycle Sequencing Kit (Thermo Fisher Scientific). The resulting PCR products were purified with the Bioanalysis NucleoSEQ Kit (Macherey–Nagel), and 15 µL of each eluate was combined with an equal volume of Hi-Di formamide (Thermo Fisher Scientific). Finally, sequencing was carried out using a 3500 Genetic Analyzer (Applied Biosystems/Hitachi, Darmstadt, Germany).

### Calculation of the minimum infection rate (MIR)

To compare the spatial and temporal differences in virus circulation, minimum infection rates (MIR) were calculated [76]. The MIR is the number of positive pools divided by the number of tested pools multiplied by 1000.

### Statistical analysis

The statistical analysis was carried out using the open-source software environment R [80]. Differences in the MIR of the individual German federal states over the monitored years from 2019 to 2022 and of North-Rhine Westphalia and Lower Saxony from 2019 to 2023 were compared by two-sided Fisher’s exact tests with Monte Carlo simulations. Differences were considered significant when *P* < 0.05. If the initial test showed a significant result, a post hoc analysis involving multiple two-sided Fisher’s exact tests was conducted to compare the year with the highest MIR with the other years. Differences were considered significant when *P* < 0.05.

### Definition of the vector year

For better comparison of virus detection in biting midges with the occurrence of disease in domestic ruminants, the respective vector year was defined to begin on 1 April and end on 31 March in the following year (Fig. 2A). This timeframe includes the acute cases of virus infections in ruminants, that can be detected within the vector season (April to October), and virus detection in aborted or malformed calves, lambs and goat kids, which occur up to 8 months after the acute infection of the pregnant dam. There are two notable exceptions from this approach: the months January to March were included in the vector year 2019/2020. Further, in 2022, midges were collected until October; therefore, the vector year 2022 comprises only the months April to October (Fig. 2A).

## Results

### Virus screening

The total number of *Culicoides* specimens collected across the study years was as follows: 226,083 in 2019 (including 4,506 males), 194,836 in 2020 (3,271 males), 580,162 in 2021 (7,990 males) and 224,282 in 2022 (4,537 males). Of all biting midges caught from 2019 to 2023, a total of 511,788 female *Culicoides* specimens were selected, morphologically identified and sorted into 19,521 pools, containing either species of the subgenus *Avaritia*, the subgenus *Culicoides* or other subgenera. All pools were tested for genome of SBV, BTV and EHDV by real-time RT-PCR. SBV genome was detected in every study year, in a total of 721 pools (3.7%). None of the pools were positive for BTV or EHDV.

**Fig. 3:**
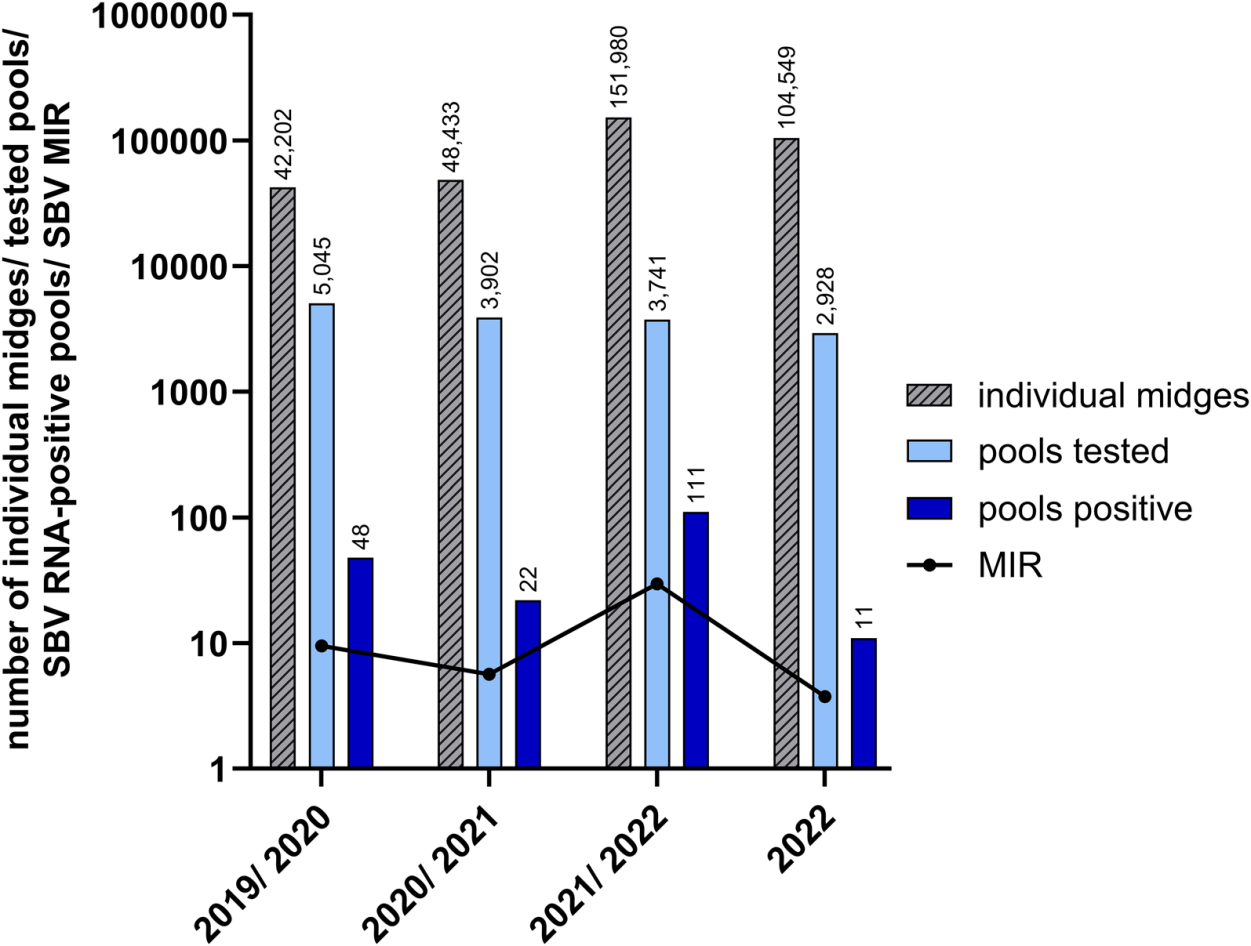
Comparison of the number of individual midges, tested pools, and Schmallenberg (SBV) RNA-positive pools over the monitored vector years. The number of individual midges is depicted by a grey bar, the total number of tested pools is depicted by a light blue bar and the number of SBV RNA-positive pools is depicted by a dark blue bar. The precise number of each group is written above each bar. To show differences of SBV circulation over the monitored vector years, the minimum infection rate (MIR) (black line) was calculated

From 2019 to 2022, midges were collected during the whole year, and from January 2019 to March 2020, a total of 42,202 biting midges from 40 trapping locations (livestock farms) were grouped into 5,046 pools. In the tested pools of 17 trapping locations, SBV RNA could be detected (Fig. 1). In total from January 2019 to March 2020, 48 pools (MIR: 9.51) tested positive for SBV RNA (Fig. 3). In the vector year 2020/2021, 48,433 *Culicoides* biting midges were processed and sorted into 3,901 pools. SBV RNA was found in a total of 22 pools (MIR: 5.64) in 15 out of 31 tested farms (Figs. 1 and 3). In the next vector year 2021/2022, 151,981 midges from 19 trapping locations were grouped into 3,741 pools. SBV RNA-positive pools were found on eight farms in a total of 111 pools (MIR: 29.67) (Figs. 1 and 3). From April to October 2022, a total of 104,549 *Culicoides* was sorted into 2,928 pools. Altogether, 11 pools (MIR: 3.76) from eight farms out of 29 tested trapping locations were positive for SBV RNA (Figs. 1 and 3).

**Fig. 4:**
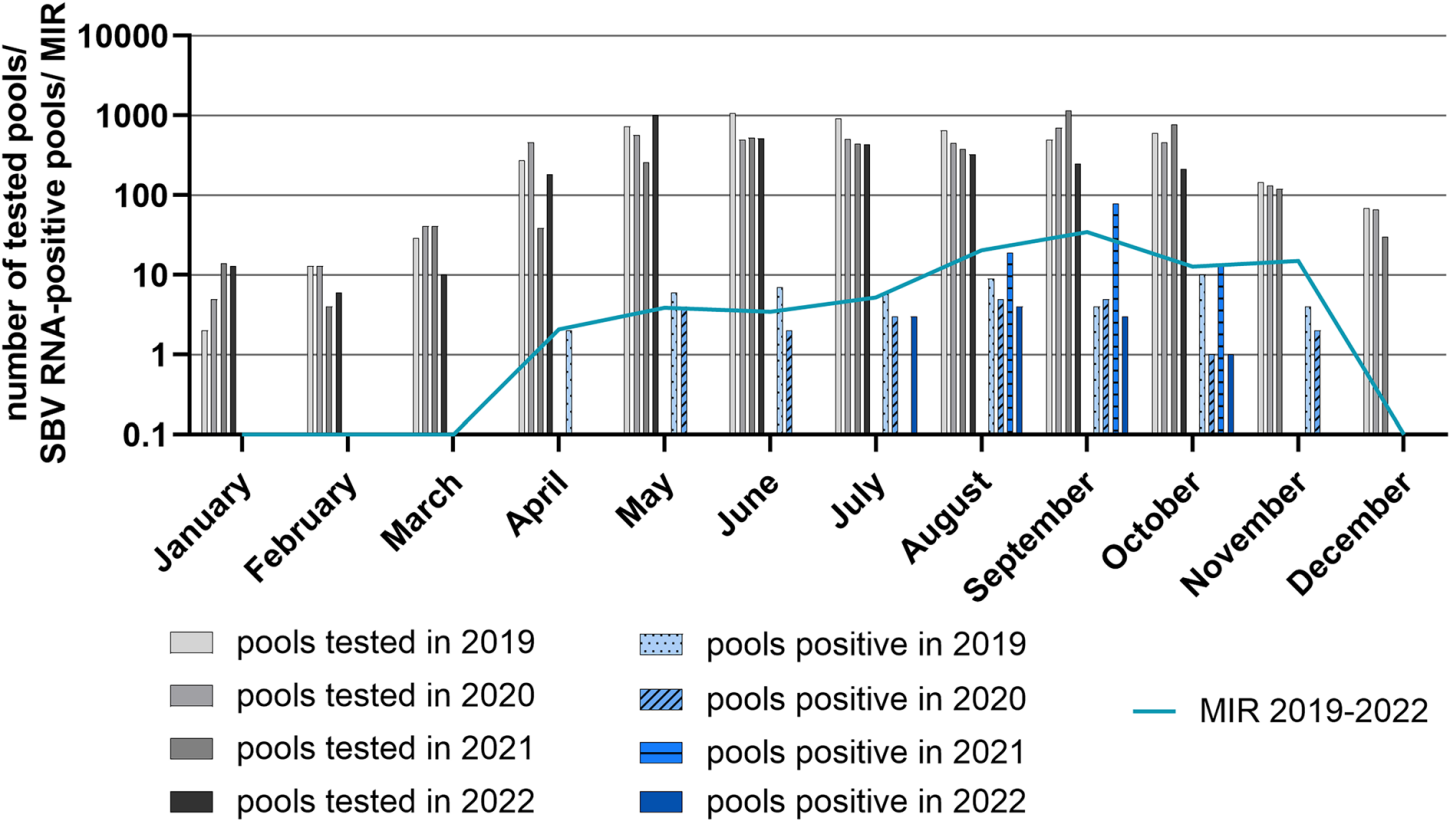
Number of tested biting midge pools and Schmallenberg virus (SBV) RNA-positive pools. For each month from 2019 to 2022, the number of tested pools is displayed in grey and the number of SBV RNA-positive pools in blue. To show monthly differences of SBV circulation over the monitored years, the minimum infection rate (MIR) (line) was calculated

Over the monitored years from 2019 to 2022 more *Culicoides* pools collected in the months from April to October were tested than collected in the months from November to March (Fig. 4). The highest rate of SBV RNA-positive pools was found in September (MIR: 34.75), followed by August (MIR: 20.43), November (MIR: 15.03) and October (MIR: 12.78), while the MIRs obtained for April (2.09), May (3.90), June (3.45) and July (5.23) were markedly lower (Fig. 4). The first SBV RNA-positive pool in a year was sampled in Beerfelde (federal state of Brandenburg) on 24 April 2019. The latest catch midges tested SBV RNA-positive was made on 21 November 2020 on the trapping site in Mühlenbach (Baden-Wuerttemberg).

**Fig. 5:**
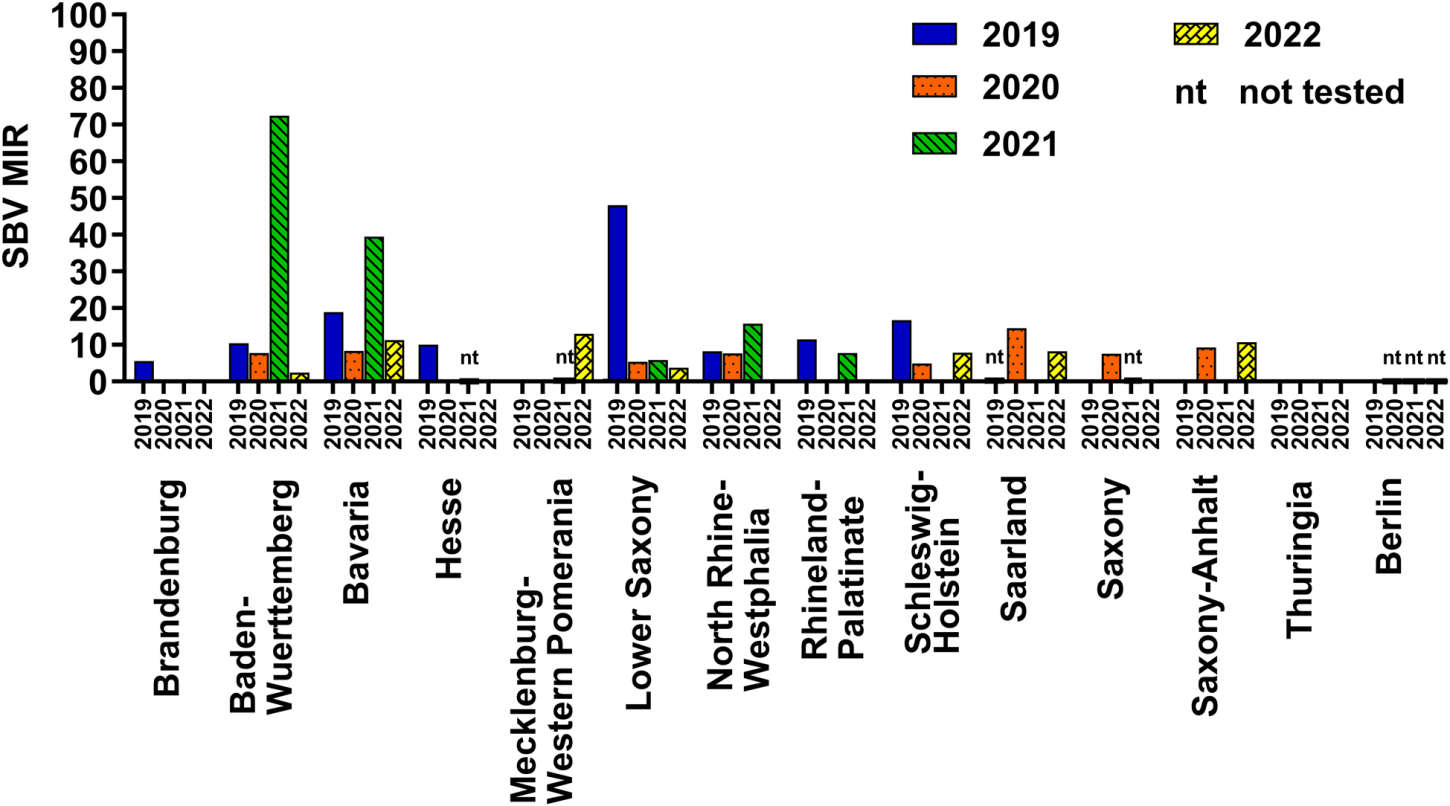
Schmallenberg virus (SBV) RNA detection in the different German federal states. To show regional differences of SBV RNA prevalence, the minimum infection rate (MIR) was calculated for the individual German federal states from 2019 to 2022

The MIR of SBV RNA in biting midge populations varied over the monitored years in the individual German federal states (Fig. 5). In 3 out of the 14 monitored German federal states, namely Lower Saxony, Baden-Wuerttemberg and Bavaria, the MIR was significantly higher in 1 year than in the other years. Statistical analysis of the MIRs in the other federal states revealed no significant differences, although in most of the states, a higher MIR is observed for at least one of the years. In Lower Saxony, the highest MIR was detected in 2019 with 48.08. In the other years, the MIR was much lower with 5.38 (2020), 5.84 (2021) and 3.77 (2022). Statistical analysis showed a highly significant difference (*P* < 0.001) in the measured MIRs in Lower Saxony, and individual comparison of the MIRs from 2019 with the MIRs from 2020, 2021 and 2022 resulted in *P*-values of *P* = 0.00238 for 2019 versus 2020, *P* = 0.00033 for 2019 versus 2021 and *P* = 0.00329 for 2019 versus 2022. A high MIR could be observed in Rhineland-Palatinate with 11.49 and in Schleswig–Holstein with 16.67 in 2019 as well. In Rhineland-Palatinate, SBV RNA was found in only one other monitored year, namely in 2021 (MIR: 7.72). In Schleswig–Holstein, the MIR was 4.93 in 2020 and 7.81 in 2022, while no SBV RNA was found in 2021. In contrast, in the southern German federal states Bavaria and Baden-Wuerttemberg, the highest MIR (39.51 and 72.45, respectively) was observed in 2021, while MIRs of 10.42 (2019), 7.72 (2020) and 2.44 (2022) were calculated for Baden-Wuerttemberg. MIRs of 19.87 (2019), 8.33 (2020) and 11.27 (2022) were determined for Bavaria in the other years. The measured high MIRs in 2021 resulted in a significant difference in Bavaria (*P* = 0.0383) and Baden-Wuerttemberg (*P* < 0.001). Individual comparison of the MIR from 2021 with the MIRs from 2019, 2020 and 2022 measured in Bavaria showed significant differences for 2021 versus 2020 with *P* = 0.0309 and 2021 versus 2022 with *P* = 0.0247, while the comparison of 2021 versus 2019 showed no significant difference. All individual comparisons of the MIR 2021 of Baden-Wuerttemberg with the MIRs of the other years revealed highly significant *P*-values of *P* < 0.001. In 2021, there was a slight increase of SBV RNA-positive pools also in North Rhine-Westphalia with a MIR of 15.78, which was higher than in the other years that had MIRs of 8.40 (2019), 7.72 (2020) and 0 (2022). In Saxony-Anhalt (Central Germany), there was an increase of the detected MIR in two of the monitored years with 9.26 in 2020 and 10.75 in 2022, while no SBV RNA was found in 2019 and 2021. In four of the German federal states, SBV RNA-positive pools were found in only one of the tested years, namely in Hesse in 2019 (MIR 10.07), in Brandenburg in 2019 (MIR 5.56), in Saxony in 2020 (MIR 7.58) and in Mecklenburg-Western Pomerania in 2022 (MIR 12.99). In two of the monitored federal states, namely Thuringia and Berlin, no SBV RNA could be detected in any of the tested biting midge pools, although it has to be taken into account that Berlin was only sampled in 2019 and not in the following years.

**Fig. 6:**
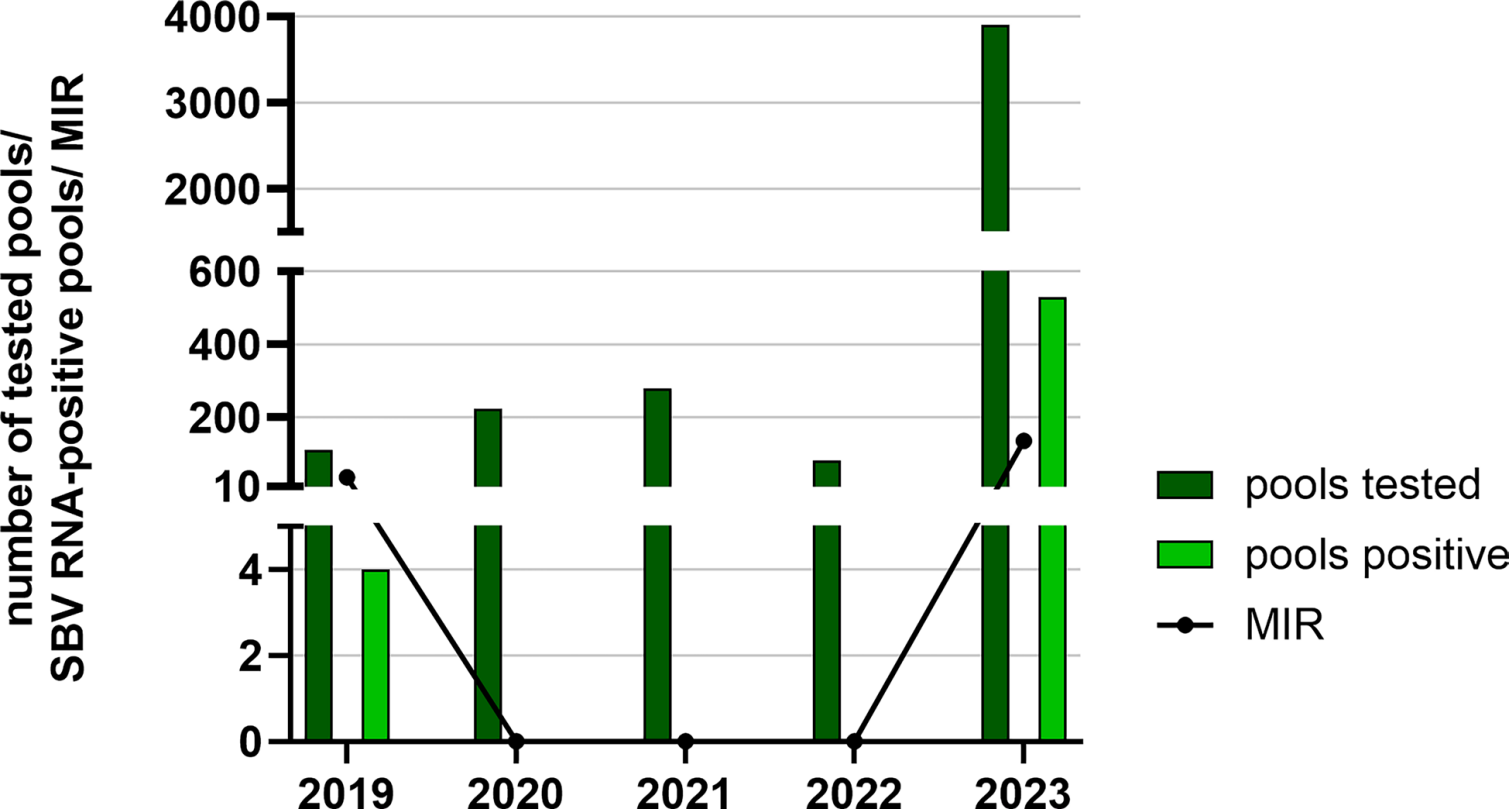
Comparison of Schmallenberg virus (SBV) RNA circulation in the west of Germany. The tested midge pools (dark green) and SBV RNA-positive pools (light green) were collected from 24 September to 12 November in 2019, 2020, 2021, 2022 and 2023 in North Rhine-Westphalia and Lower Saxony. To show the differences in SBV RNA occurrence, the minimum infection rate (MIR) in the midges was calculated for each monitored year

In 2023, 164,623 biting midges were caught from the end of September to the beginning of November on 13 farms located near the German–Dutch border. The midges were grouped into 3,905 pools, and SBV RNA-positive pools could be detected on every tested livestock farm except for one (Fig. 1). Since many pools were tested positive for SBV RNA in 2023, the results of this timeframe were compared with the results of the corresponding timeframe and area in the other monitored years (Fig. 6). In 2023, SBV RNA was found in 529 out of 3,905 tested pools (MIR: 135.47). In the same federal states and in the respective time period in 2019, SBV RNA was found in 4 out of 110 tested pools (MIR: 36.36), while in 2020, 2021 and 2022, no SBV RNA was detected in any of the pools collected in North Rhine-Westphalia and Lower Saxony from the end of September to the beginning of November. Statistical analysis of comparison of the SBV MIRs measured from 2019 to 2023 in North-Rhine Westphalia and Lower Saxony revealed a highly significant difference, with *P* < 0.001. All the individual comparisons of 2023 with 2019, 2020, 2021 and 2022 resulted in highly significant *P*-values (*P* < 0.001) as well.

**Fig. 7:**
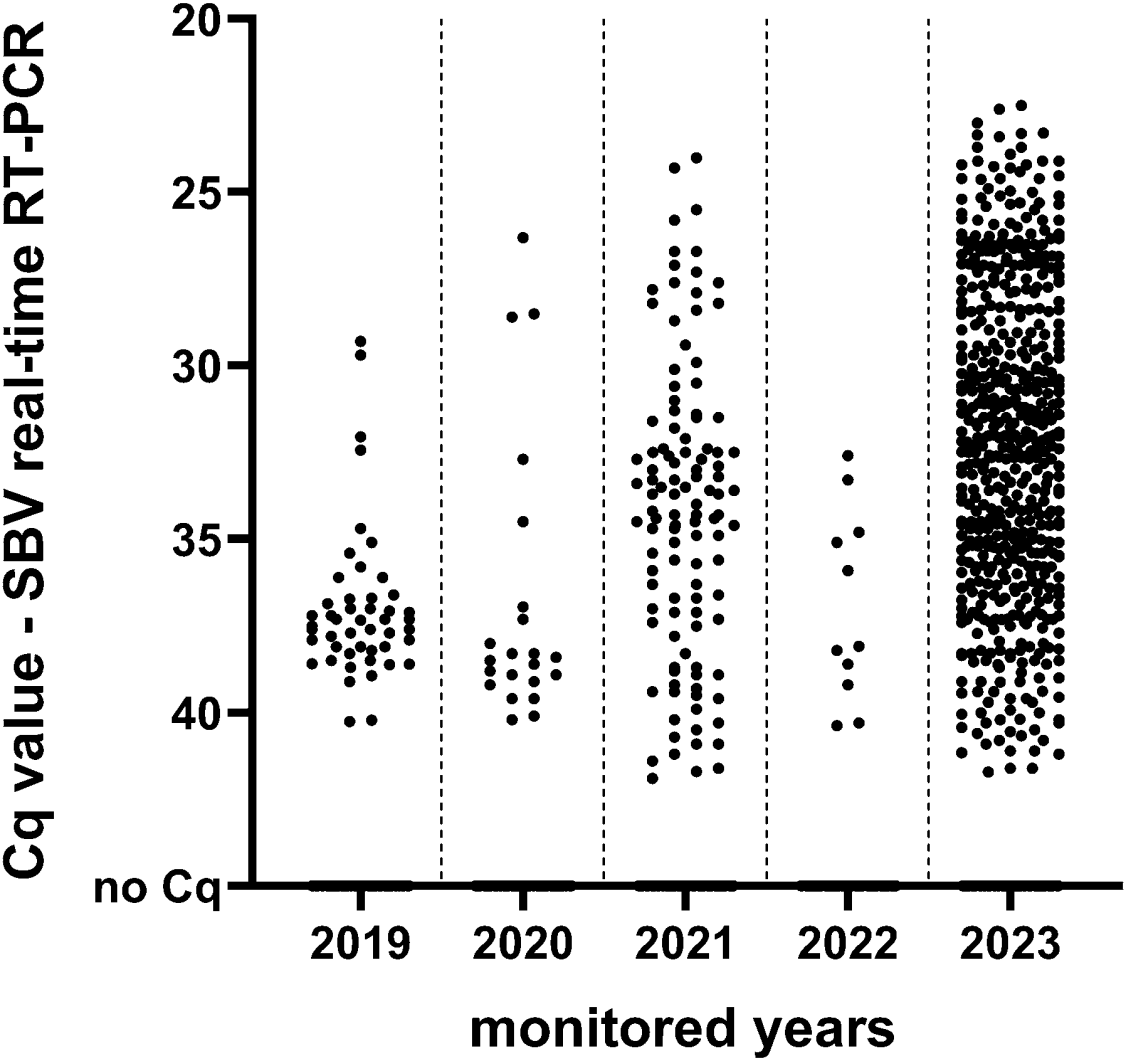
Distribution of quantification cycle values (Cq values) resulting from the Schmallenberg virus (SBV) real-time RT-PCR. The Cq values of the tested biting midge pools are depicted for each monitored year from 2019 to 2023

**Fig. 8:**
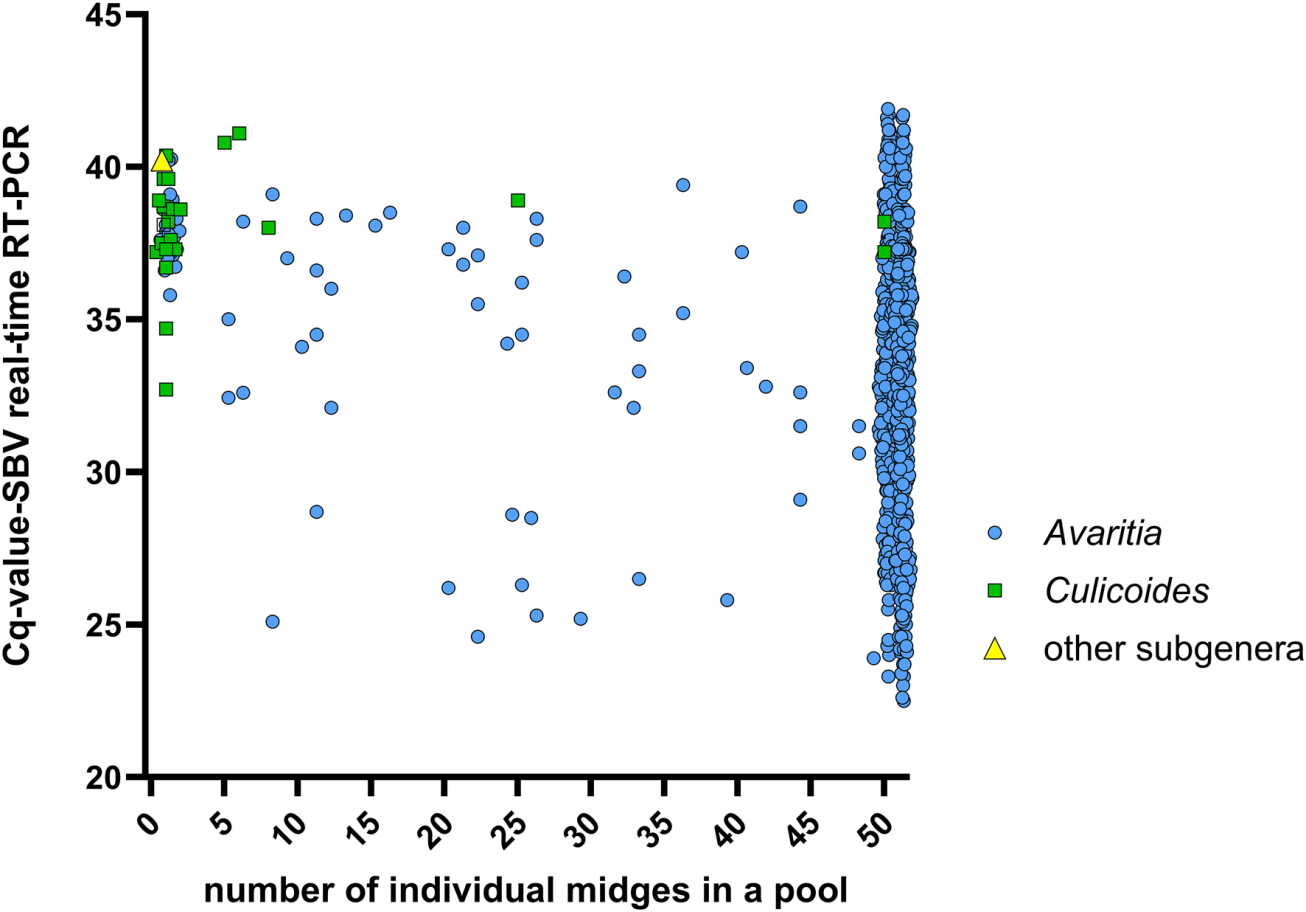
Number of individual midges per pool in relation to the quantification cycle values (Cq value) of the Schmallenberg virus (SBV) real-time RT-PCR. The subgenus *Avaritia* is depicted by blue circles, the subgenus *Culicoides* is depicted by green squares and other subgenera are depicted by yellow triangles

The majority of all SBV RNA-positive pools from 2019 to 2023 showed quantification cycle values (Cq values) above 30. The percentage of the positive pools with Cq values below 30 was 4.1% in 2019, 13.6% in 2020, 16.2% in 2021, 0% in 2022 and 31.0% in 2023. In 2019, the Cq values ranged from 29.3 to 40.3, in 2020 from 26.3 to 40.2, in 2021 from 24.0 to 41.9, in 2022 from 32.6 to 40.4 and in 2023 from 22.5 to 41.7 (Fig. 7). The lowest Cq values (< 25) were found in pools of 50 midges from the subgenus *Avaritia* (Fig. 8). Only two *Avaritia* pools with less than 15 midges showed Cq values below 30 (Fig. 8). The pools of the subgenus *Culicoides* and other subgenera averagely contained less midges but showed high Cq values above 30 (Fig. 8).

### *Culicoides* identification

**Fig. 9:**
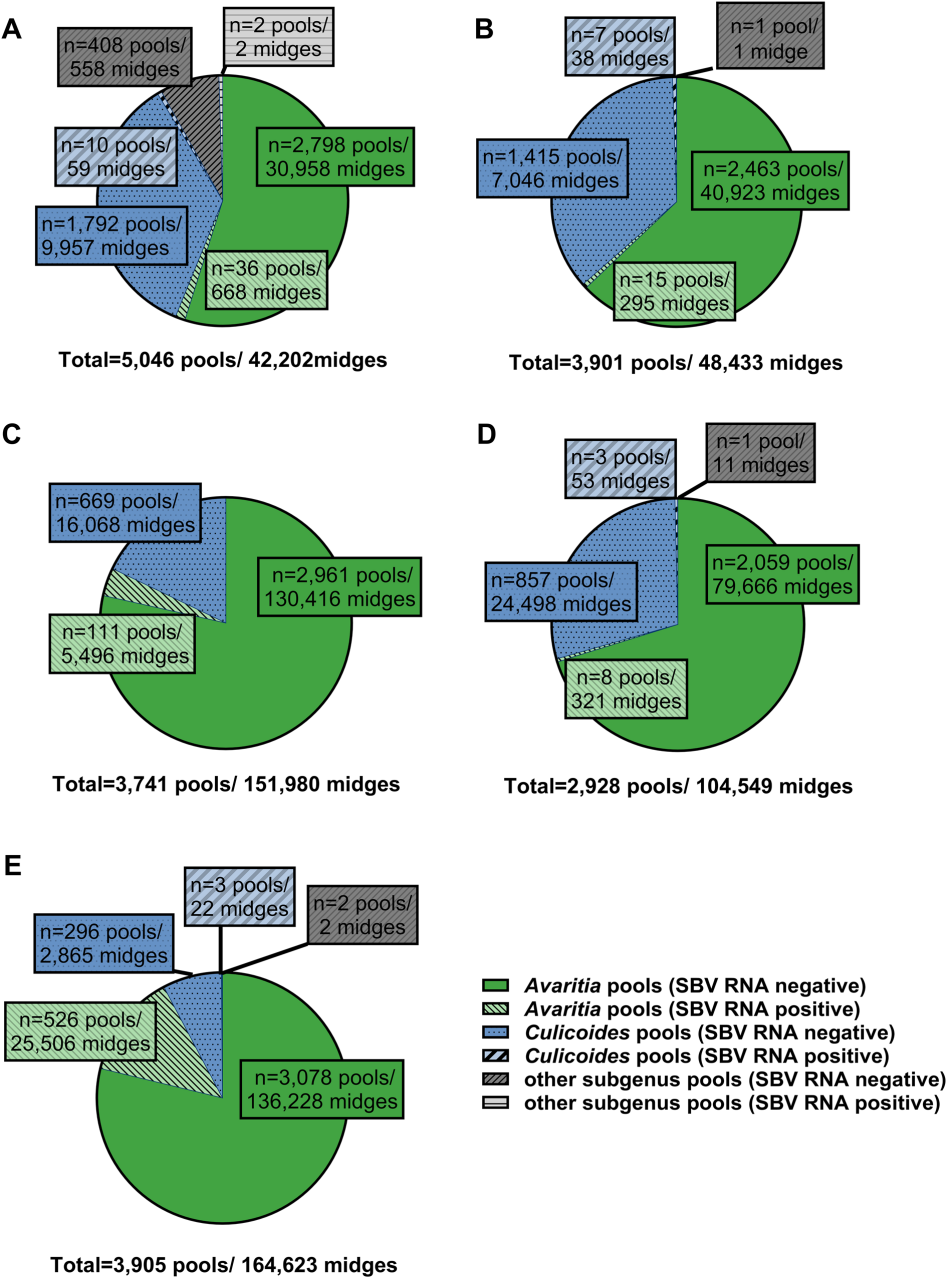
Proportion of tested pools and specimens of the subgenus *Avaritia*, subgenus *Culicoides* and other subgenera as based on morphological identification. The results of the of Schmallenberg virus (SBV) RNA-positive and -negative pools from the vector year 2019/2020 are depicted in **A**, the vector year 2020/2021 in **B**, the vector year 2021/2022 in **C**, the vector year 2022 in **D** and the period from September to November 2023 in **E**. Absolute numbers are displayed in the individual figures

For this study, 511,788 female *Culicoides* midges caught from 2019 to 2023 were morphologically identified. The majority of 450,607 individual midges was assigned to the subgenus *Avaritia* and placed in 14,055 pools (72.0%) with an average pool size of 32.3 midges. A total of 60,606 specimens were identified as subgenus *Culicoides* and pooled in 5,052 pools (25.9%) with a mean pool size of 12.0 midges. Only 574 *Culicoides* midges were assigned to species of other subgenera and put in 414 pools (2.1%) with an average of 1.4 midges per pool. The detailed results of the morphological identification of the tested *Culicoides* biting midges for every monitored year are displayed in Fig. 9 and Table 1.

**Table 1:** Number of *Culicoides* morphologically identified in every vector year

Vector year	Subgenus	Individual midges	Tested pools	Average pool size	SBV RNA positive pools
2019/2020	*Avaritia*	31,626	2,834 (56.2%)	11.2	36 (1.3%)
	*Culicoides*	10,016	1,802 (35.7%)	5.6	10 (0.6%)
	Other subgenera	560	410 (8.1%)	1.4	2 (0.5%)
		42,202	5,046	8.4	48 (1.0%)
2020/2021	*Avaritia*	41,348	2,478 (63.5%)	16.7	15 (0.6%)
	*Culicoides*	7,084	1,422 (36.5%)	5.0	7 (0.5%)
	Other subgenera	1	1 (0.03%)	1.0	0 (0.0%)
		48,433	3,901	12.4	22 (0.6%)
2021/2022	*Avaritia*	135,913	3,072 (82.1%)	44.2	111 (3.6%)
	*Culicoides*	16,068	669 (17.9%)	24.0	0 (0.0%)
	Other subgenera	0	0 (0.0%)	0.0	0 (0.0%)
		151,981	3,741	40.6	111 (3.0%)
2022	*Avaritia*	79,987	2,067 (70.6%)	40.9	8 (0.4%)
	*Culicoides*	24,551	860 (29.4%)	28.9	3 (0.3%)
	Other subgenera	11	1 (0.03%)	11.0	0 (0.0%)
		104,549	2,928	37.2	11 (0.4%)
2023	*Avaritia*	161,734	3,604 (92.3%)	44.9	526 (14.6%)
	*Culicoides*	2,887	299 (7.7%)	9.7	3 (1.0%)
	Other subgenera	2	2 (0.05%)	1	0 (0.0%)
		164,623	3,905	42.2	529 (13.6%)

The midges morphologically identified as subgenus *Avaritia*, subgenus *Culicoides* or other subgenera, the respective pool numbers and the SBV RNA positive pools are displayed in total numbers. The percentages in the brackets refer to the total number of pools tested in the respective vector year. Additionally, the average pool size was calculated for the pools

In every monitored year, the most SBV RNA-positive pools were represented by the subgenus *Avaritia* (Fig. 9 and Table 1). The respective percentages of SBV RNA-positive pools of the subgenus *Culicoides* were lower (Fig. 9 and Table 1). In all monitored years, only two pools with morphologically incorrectly identified other *Culicoides* subgenera from the year 2019 were found positive for SBV RNA.

In the species-specific PCR for the subgenus *Avaritia*, which was used to identify the species composition in the SBV RNA-positive pools, the most abundant detected species were *C. obsoletus* and *C. scoticus*. Several pools additionally contained the species *C. dewulfi* and *C. chiopterus.* In the PCR for the differentiation of the haplotype/clade of *C. obsoletus*, the clade O1 was most often detected. In the species-specific PCR for the SBV RNA-positive pools of the subgenus *Culicoides*, *C. punctatus* was most often found, followed by *C. pulicaris.* One pool that was morphologically determined as *C. newsteadi* was found to be *C. selandicus* by sequencing. The two SBV RNA-positive pools of other species were sequenced, and the pool of *C. circumscriptus* was found to be *C. griseidorsum*, and *C. riethi* was identified as *C. lupicaris* haplotype L2. The detailed results of the species identification are listed in the Supplementary Dataset in the Zenodo repository (10.5281/zenodo.15083498).

## Discussion

In our monitoring programme, we collected data on SBV, BTV and EHDV occurrence in *Culicoides* biting midges all over Germany for five consecutive years (2019–2023). The investigated period of 5 years and the nationwide collection of midges make it possible to follow the spatial and temporal differences in the occurrence of the viruses in the midges. It should be noted that the investigated viruses may cause subclinical diseases in adult ruminant hosts such as cattle, which nevertheless can lead to economic losses, e.g. due to increased abortion rates or reduced milk yields. Because it is often difficult to detect the diseases in ruminants, the detection of the viruses in the midge vector is a suitable alternative to retrospectively monitor the occurrence of SBV, BTV and EHDV.

Our results confirm an annual circulation of SBV in biting midges, while neither BTV nor EHDV were found. We could detect SBV genome every year in the biting midges, although with variations in the regional and seasonal distribution. When comparing the detected MIRs, it should be taken into account that the calculation assumes only one positive midge in a pool, which may not reflect reality. The more midges are pooled together, the higher the probability that more than one positive midge is included [17]. From 2019 to 2022, biting midges were caught year-round, including the winter months. We found SBV RNA in pools of biting midges caught from April to November and none in the months from December to March. Most of the SBV RNA-positive pools were collected in August and September, although a high proportion of pools tested positive also in November. Other monitoring studies in Europe found similar results. The majority of the studies from Belgium, Netherlands, Denmark, Poland, France, Spain and Italy were conducted in summer or autumn and found an increased SBV circulation from August to October [40, 41, 58, 81–83]. Studies that included *Culicoides* sampling in the winter months were conducted in Italy and Belgium. In Italy, biting midges were caught from June 2011 to June 2012. SBV RNA-positive pools were found from September to October 2011 and in May 2012 [84]. In Belgium, midges were collected from January to December 2011, with pools from August to October 2011 found SBV RNA-positive [38]. Overall, these former studies are not easy to compare. They vary in pool size, period of biting midge collection, placement of the traps and patterns of the midge collection from the traps. Furthermore, the studies differed in the evaluation of the positive pools, with calculations of variable parameters and separated results of the different *Culicoides* species or trapping regions. In Germany, only one other study investigating SBV circulation in the biting midges was conducted from 2011 to 2014 [49]. In this study, 945 pools with a total of 21,397 midges collected all over Germany were screened for SBV RNA. Only two pools (0.2%) caught in summer 2012 were found positive, although there was a massive outbreak of SBV in domestic ruminants. A conceivable reason for the detected low percentage of SBV RNA-positive pools in 2012 is that the biting midges were caught on trapping sites that had initially not been selected to be used for SBV screening, and traps had been placed on farms not necessarily keeping ruminant host animals. The chance to find SBV on these untargeted trapping sites obviously was small. From the results of our monitoring and of other studies [40, 47, 58, 76, 81, 82, 84, 85], we conclude that the risk of SBV transmission in Europe is especially high in late summer and early autumn. First and foremost, in mild climatic regions, more biting midges are active in these seasons [56]. The biting midge species of the subgenus *Avaritia* and the subgenus *Culicoides* are mostly multivoltine, which means that they have multiple generations from spring to autumn and one generation overwintering as larvae in mild climatic regions such as Germany [86]. Usually two peaks of adult activity are visible over the course of a year, one peak in spring after the development of the overwintering larvae and a second, even larger peak in late summer or early autumn after the emergence of the imagines of the second generation [87]. Another reason for the effective SBV circulation in the biting midge population in summer and early autumn is the improved chance of virus transmission due to temperatures above 25 °C. This was mainly described for orbiviruses in laboratory colonies of *C. sonorensis* [88–91]. A similar correlation of temperature and transmission of SBV under natural conditions is very likely [92]. The highest average temperatures in Germany from 2019 to 2022 were measured during the summer from June to August (source: Deutscher Wetterdienst).

The abundance of native biting midges and the chance of virus transmission have been shown to be reduced in winter owing to slower development of midges at low temperatures [42]. Several studies indicated that the threshold temperature of virus replication and transmission in the *Culicoides* vector is about 12 °C [35, 88, 93]. In Germany, an average temperature below this threshold (12 °C) was observed in the months from October/November to April/May from 2019 to 2022 (source: Deutscher Wetterdienst). In our study, in the winter months from December to March, fewer biting midges were caught, and, therefore, less midges were screened for viruses than in other seasons, with no SBV RNA being found in any of the tested pools. However, it should be considered that fewer biting midges could be caught during the winter than in summer because less UV-traps had been operated. Nonetheless, in comparison with the number of biting midges caught from April to November, the number of caught midges decreased remarkably in the time from December to March. Therefore, the risk of virus transmission seems to be drastically reduced during the winter months.

In addition to the differences of SBV circulation over the course of the year, we found spatial variations over the monitored years. When taking a closer look at the detected MIRs in the individual German federal states from 2019 to 2022, it becomes apparent that a high level of SBV circulation cannot be stated for all federal states equally. Rather, there are regional peaks of SBV RNA-positive pools in different years. These annual peaks are especially apparent, as well as statistically significant in the federal states of Baden-Wuerttemberg (2021), Bavaria (2021) and Lower Saxony (2019); however, in almost all the other federal states, except for Thuringia and Berlin (which was included only in 2019), an increased MIR could be observed for at least one of the monitored years from 2019 to 2022. In 2023, a statistically significant high SBV RNA occurrence level could again be detected in North Rhine-Westphalia and Lower Saxony. However, it must be considered that no other federal states were tested in 2023. On the basis of these findings, it can be concluded that there was a high SBV circulation in 2019 in the northwest of Germany, in 2021 in the south of Germany and in 2023 most notably in the west of Germany. We hypothesize that the variation in the levels of SBV circulation is a consequence of the cyclic re-circulation pattern of SBV in ruminants [3, 94, 95]. After the first outbreak of SBV in 2011, many European countries observed a re-circulation of SBV to a larger extent in domestic ruminants every 2 to 4 years [3, 94–97]. In general, the risk of SBV outbreaks is expected to increase as seroprevalence in the ruminant population gradually decreases over time. Though natural SBV infection leads to the production of long persisting antibodies [98], the herd seroprevalence is gradually declining over time owing to the slaughter of immunoprotected animals, the rapid decrease of maternal antibodies of newborn animals and the import of naïve animals [98]. In the years of low infection prevalences in ruminants, the transmission of virus to biting midges during a blood meal is less likely. In contrast, in years of extensive SBV circulation in the mammalian host animals, the chances of virus transmission to the biting midges increase. Evidence of annual fluctuations of SBV occurrence in biting midge populations can be found also in former published SBV monitoring studies from other European countries. In Belgium and the Netherlands, the first outbreak had its peak in 2011, leading to relatively high SBV prevalences in the midges, with 6.7% in Belgium (with up to 25 midges/pool) and 2.3% in Netherlands (with 10 midges/pool) [83, 85]. The consequence was a higher proportion of immunoprotected ruminants and therefore reduced SBV circulation in the biting midge population in 2012 with SBV prevalences of 3.6% in Belgium (with a mean of 19.3 midges/pool) and 1.5% in Netherlands (50 midges/pool) [82, 99]. In Denmark, SBV also arrived in 2011, though later in the year than in Central Europe, and therefore circulated again at a higher extend in 2012 [81], with a detected SBV prevalence of 9.1% in 2011 (5 midges/pool) and an even higher prevalence of 15.8% in 2012 (with a mean pool size of 6.5 midges/pool) [81, 100]. In 2016, extensive SBV re-circulation in biting midges and domestic ruminants was detected in Belgium and Poland, confirming the idea of SBV recurrence in midges when immunoprotected ruminants are scarce [76, 101]. In our study, we saw a similar fluctuation of SBV circulation in the biting midge population in different regions of Germany. According to our findings in the midges, we should therefore have seen a high SBV prevalence in the domestic ruminants in 2021 in the south and in 2023 in the west of Germany. In fact, there was no noticeable increase of reported SBV cases in ruminants; this may be a consequence of a massive under-detection and under-reporting [94]. However, from September 2021 to January 2022, a study searching for SBV in wildlife in five federal states of Germany found SBV antibodies in feral ruminants, especially in Hesse, Rhineland-Palatinate and, to a lesser extent, in North Rhine-Westphalia, while no antibodies were found in Bavaria and Mecklenburg-Western Pomerania [4]. Unfortunately, we did not test any biting midges from 2021 from Hesse; however, in the same year, we detected SBV RNA-positive midges in North Rhine-Westphalia and Rhineland-Palatinate. In accordance with our findings of high MIRs in the *Culicoides* population in North-Rhine Westphalia and Rhineland Palatinate in 2023, a high seroprevalence of over 40% was found in wild ruminants in 2023 in North-Rhine Westphalia which proves extensive SBV circulation at that time [102].

In contrast to SBV, we could not find any EHDV or BTV in the tested *Culicoides* pools from 2019 to 2022. The absence of EHDV genome in biting midges is in line with the epidemiological situation in ruminants in Germany. However, since the first introduction of EHDV in the south of Europe in 2022, the virus spread northwards, leading to the detection of the first ruminant EHDV cases in France in September 2023 [103]. Although in 2024 the spread of EHDV through France was stopped, with a limited occurrence of EHDV in the south and northwest of the country (source: ESA), a further spread of EHDV in Europe with an emergence in Germany cannot be ruled out in the next years.

As was the case for EHDV, BTV was not detected in the period from 2019 to 2022. However, the epidemiological situation of BTV in Germany is different from that for EHDV. After Germany was free of BTV from 2012 to December 2018, BTV-8 re-occurred in 2019 with 59 cases in domestic ruminants in the southwest of the country (source: TSN). In 2020 and 2021, three cases were reported, and in 2022, no cases of BTV-8 were reported (source: TSN). According to the data from the German animal disease reporting system, we suppose that BTV-8 circulation in the biting midge population from 2019 to 2022 was on a low level, causing only regional outbreaks with a very limited number of cases, not comparable with the massive outbreak seen in 2006/2007. With the results of our study, we confirmed this very low BTV circulation also in the biting midge population. After the emergence of BTV-3 in 2023 in the Netherlands and the rapid spread within the country and to the west of Germany, we searched for BTV-3 positive biting midge pools in the affected regions in North Rhine-Westphalia and Lower Saxony. Again, none of the tested pools were positive for BTV genome. However, a part of the biting midge pools in our monitoring programme that had been caught in autumn 2023 was analysed prior to this study. In this preceding virus screening, we were able to find the BTV-3 genome in one of the tested biting midge pools [17], confirming that BTV-3 circulated on a very low level in the west of Germany in autumn 2023. Likewise, BTV-3 was only detected in a few ruminants in the west of Germany (source: TSN). Cold temperatures stopped its circulation in the biting midges and prevented further spreading until early 2024. However, in spring, summer and autumn 2024, BTV-3 continued to circulate, at that time on a much higher extent with a massive outbreak in ruminants all over Germany (source: TSN). The dynamic disease situation in ruminants in Europe makes it essential to continue the monitoring and the virus screening in the *Culicoides* biting midges.

Another aim of our monitoring programme was to find out which *Culicoides* species are possibly involved in virus transmission. The morphologically determined *Culicoides* species of all monitored years belonged almost exclusively to the subgenus *Avaritia* and the subgenus *Culicoides*. The species of these two subgenera are the most abundant in Europe [104] and were therefore more frequently collected and tested for viruses than other *Culicoides* species. In our study, only a small share of caught biting midges was identified as other species, resulting in a possibly biased detected prevalence of viruses. The finding that mainly biting midges of the subgenus *Avaritia* were infected with SBV matches the outcome of other European studies [41, 42, 48, 59, 76, 100]. By further differentiating the morphologically presorted subgenera by molecular biological methods, it was possible to narrow down the putative vector species. The SBV RNA-positive subgenus *Avaritia* pools were further differentiated into the taxa *C. obsoletus* clade O1, *C. obsoletus* clade O2, *C. scoticus*, *C. dewulfi* and *C. chiopterus*. The SBV RNA-positive subgenus *Culicoides* pools contained *C. punctatus* and *C. pulicaris*. One single midge, which was found in the north of Germany and tested positive, was identified by sequencing as *C. selandicus*. This species belongs to the subgenus *Culicoides* and was thought to be mainly found in Denmark [105] but has recently been recorded in Sweden, Slovakia, the UK and Germany [51]. The SBV RNA-positive pools that had been morphologically incorrectly identified as *C. circumscriptus* and *C. riethi* were sequenced and found to be *C. griseidorsum* and *C. lupicaris* L2, respectively. Genetic identification is much more reliable than morphological identification since clear data are generated that are not prone to subjective misinterpretation. *Culicoides lupicaris* is a species of the subgenus *Culicoides*. Although SBV has already been found in this species in previous studies [40], this is the first time that an SBV-positive *C. lupicaris* midge could be specified as haplotype L2.* Culicoides griseidorsum* is a species within the subgenus *Sensiculicoides* Shevchenko [106]. While this is the first detection of SBV genome in the species *C. selandicus* and *C. griseidorsum*, no information is available about their vector competence for SBV. The midges might just have contained a residual blood meal from a viremic host without facilitating virus replication.

The detection of SBV genomes in biting midges in the field is not sufficient evidence for vector competence unless the virus is detected in the salivary glands. In our study, the tested midges were analysed as whole individuals. With the usage of the whole midge for virus detection, it is not possible to confirm whether the virus was present in the digestive track only or disseminated in the body and also spread to the salivary glands. Only when the virus reaches the salivary glands, the midge is able to transmit the virus when taking a blood meal from a naïve host [76]. In addition, up to four different taxa were found in some SBV RNA-positive pools by the species-specific PCRs. In these cases, it is not possible to determine which of the species might be the putative vector. Vector competence studies that demonstrate the ability of *Culicoides* species to replicate and transmit a virus are scarce. All of the SBV RNA-positive pools with biting midges of the subgenus *Culicoides* and other species had rather high Cq values of over 32. By contrast, we found several SBV RNA-positive pools containing the midge taxa *C. obsoletus* clade O1, *C. dewulfi*, *C. chiopterus* and *C. scoticus* with Cq values under 32. From infection studies with SBV in laboratory colonies of biting midges it is known that midges with high virus replication rates and therefore low Cq values (< 24 or < 32) could carry transmissible infections [58, 107, 108]. However, Cq values under 32 may be detected as well when the midges were freshly fed on a viremic host. Additionally, we have to take in account that a pool consisted of up to 50 midges, including the possibility that more than one midge was virus-positive. Therefore, the most informative pools might be the pools containing only a few individual midges and displaying low Cq values in the SBV-specific PCR. In our study, this applies only to a few pools with midges of the subgenus *Avaritia*. In a pool of eight midges with a Cq value of 25.1, the included species were found to be *C. obsoletus* clade O1 and *C. chiopterus*, and in a pool of 11 midges with a Cq value of 28.7, the species were identified as *C. scoticus*, *C. obsoletus* clade O1 and *C. chiopterus*. From our results, we conclude that several pools of the subgenus *Avaritia* possibly contained biting midges with transmissible infections.

In the distribution of the Cq values in the SBV RNA-specific real-time RT-PCR, it becomes apparent that there were more pools in 2023 with Cq values below 32 than in the years before. Overall, it is possible that in 2023 more biting midges replicated SBV to a transmissible level than in the previous years. However, the storage duration of the pools in ethanol may also play a role for the detected Cq values. In 2023, the RNA extraction was done immediately after collection and species differentiation. In the years before, the pools had been stored in 80% ethanol at room temperature for several months or even years. We suspect that, to a certain degree, the RNA degrades during the long-time storage in the alcohol. In a previous study, the long-term preservation of viral RNA at room temperatures was evaluated for situations when storage at low temperatures was not possible, e.g. for collection and transport. There was no loss of viral RNA detectable at the end of the experiment after the storage of infected mosquitoes for 8 weeks in 99.5% ethanol [109]. However, in our monitoring study, the pooled midges from 2019 to 2022 had been usually stored longer than 8 weeks in 80% ethanol. Therefore, further studies evaluating the storage of the midges in ethanol at room temperature for longer than 8 weeks are required.

In summary, we showed SBV circulation in the biting midges in Germany in every monitored year, although on a different extent, depending on the region, the season and the year of midge sampling. Altogether, our study allowed the retrospective detection of circulating viruses in the biting midge population and the identification of potential vector species. We could confirm the species of the Obsoletus complex, as well as the two species of the subgenus *Avaritia C. chiopterus *and *C. dewulfi*, as the main putative vectors of SBV, enabling its annual circulation. Further continuation of the study will distinguish the role of other species, e.g. *C. punctatus*, *C. pulicaris*, *C. lupicaris*, *C. griseidorsum* and *C. selandicus*, for the transmission of SBV and other viruses.

## Conclusions

The results from the monitoring programme confirm an enzootic circulation of SBV in the biting midge population in Germany during summer and autumn. Additionally, the ongoing outbreak of BTV-3 in Europe and the emergence of EHDV in southern Europe threaten the domesticated ruminants and the agricultural sector at large. Therefore, continuation of the monitoring programme is indispensable.

## Data Availability

The datasets generated and analysed during the current study are available in the Zenodo repository, 10.5281/zenodo.15083498.

## Abbreviations

SBV: Schmallenberg virus
BTV: Bluetongue virus
EHDV: Epizootic haemorrhagic disease virus
MIR: Minimum infection rate
PCR: Polymerase chain reactions
RT-PCR: Reverse transcription polymerase chain reactions
TSN: Tierseuchen-Nachrichtensystem (German animal disease reporting system)
Cq value: Quantification cycle value
LED: Light-emitting diode
UV: Ultraviolet
